# Thrombosis risk in sickle cell disease with HIV co-infection: unraveling the complex interactions and clinical implications – a narrative review

**DOI:** 10.1097/MS9.0000000000003533

**Published:** 2025-07-10

**Authors:** Emmanuel Ifeanyi Obeagu

**Affiliations:** Department of Biomedical and Laboratory Science, Africa University, Zimbabwe

**Keywords:** hemostasis, HIV co-infection, inflammation, sickle cell disease, thrombosis risk

## Abstract

Thrombosis is a significant, yet often under-recognized, complication in individuals with co-infection of sickle cell disease (SCD) and human immunodeficiency virus (HIV). Both diseases independently elevate thrombotic risk, but their combined presence creates a complex pathophysiological environment that exacerbates endothelial dysfunction, inflammation, and procoagulant states. In SCD, sickled red blood cells cause vaso-occlusion and endothelial injury, while HIV contributes to chronic immune activation, dysregulated hemostasis, and increased thrombotic potential. The dual burden of these conditions not only increases the incidence of thrombotic events such as deep vein thrombosis, pulmonary embolism, and stroke, but also complicates their management due to overlapping symptoms and treatment challenges. This review explores the underlying mechanisms that contribute to the heightened risk of thrombosis in patients with both SCD and HIV, focusing on the interaction between these conditions at the molecular, cellular, and systemic levels. The impact of HIV on vascular health, in conjunction with the pathological effects of SCD on blood flow and clotting, creates a synergistic thrombotic risk. Furthermore, the review examines the clinical manifestations, diagnostic challenges, and treatment strategies for managing thrombosis in this co-infected population. Given the potential for significant morbidity and mortality, timely and effective management of thrombotic complications is critical.

**HIGHLIGHTS**

- Hypercoagulability: Sickle cell disease (SCD) and HIV both promote a prothrombotic state, increasing clot formation risks.
- Endothelial dysfunction: Chronic inflammation and endothelial injury in SCD and HIV exacerbate vascular damage.
- Platelet activation: HIV-driven immune activation and SCD-induced hemolysis enhance platelet aggregation.
- Inflammatory cascade: Elevated cytokines (TNF-α, IL-6) amplify clotting potential.
- Antiretroviral therapy impact: Some ARTs may further increase thrombotic risk in co-infected individuals.

## Introduction

Sickle cell disease (SCD) is a genetic blood disorder that primarily affects individuals of African descent, leading to chronic hemolysis, vaso-occlusion, and organ damage. The disease is characterized by the abnormal polymerization of hemoglobin S, which causes red blood cells to adopt a sickle shape, resulting in impaired blood flow and frequent painful crises. SCD is associated with a range of complications, including stroke, organ damage, and an increased risk of thromboembolic events. The presence of human immunodeficiency virus (HIV) co-infection adds another layer of complexity, as HIV induces chronic inflammation, immune dysregulation, and endothelial dysfunction, all of which can contribute to a heightened thrombotic risk. Thrombosis, a condition in which abnormal blood clotting leads to vessel obstruction, is a known complication in both SCD and HIV, but the combined effects of these two conditions significantly amplify the risk^[1,2]^. The impact of HIV on the vasculature is multifactorial, with HIV-related chronic inflammation and immune activation playing key roles in altering the hemostatic balance. HIV induces endothelial dysfunction, increases the production of pro-inflammatory cytokines, and impairs the regulation of coagulation. On the other hand, in SCD, the sickling of red blood cells leads to endothelial injury, increased blood viscosity, and a procoagulant state, which further exacerbates the risk of clot formation. These combined effects can lead to both venous and arterial thromboembolic events, including deep vein thrombosis (DVT), pulmonary embolism (PE), and stroke, which are common in individuals with SCD. The underlying pathophysiology of thrombosis in SCD and HIV co-infection remains poorly understood, but the interaction between these diseases at the molecular and cellular levels creates a unique clinical challenge for clinicians^[3,4]^. Given the overlapping risks and complex mechanisms that contribute to thrombosis in SCD and HIV, it is crucial to understand the specific factors that increase the likelihood of thrombotic events in this co-infected population. Chronic inflammation, altered platelet function, endothelial damage, and the use of antiretroviral therapy (ART) all contribute to a heightened thrombotic risk. ART, while critical in controlling HIV, has been associated with adverse metabolic effects, including dyslipidemia and insulin resistance, which further increase the risk of cardiovascular events. Moreover, the chronic pain, blood transfusions, and episodes of vaso-occlusive crises seen in SCD patients may further complicate the picture, as these factors can promote additional thrombotic risk. Thus, the management of thrombosis in individuals with both SCD and HIV requires a multifaceted approach that accounts for the complexity of these two co-existing conditions^[5]^.

The prevalence of thrombosis in individuals with HIV and SCD co-infection is increasing, but it remains largely underrecognized in clinical practice. The clinical manifestations of thrombosis, such as pain, swelling, and shortness of breath, may overlap with common SCD complications, making it difficult to distinguish between thrombotic events and other acute episodes of the disease. Additionally, the use of anticoagulants in these patients must be carefully managed, as the bleeding risks associated with SCD – such as hemolysis and the potential for splenic dysfunction – complicate the decision-making process. Therefore, a high index of suspicion and prompt diagnostic evaluation, including imaging techniques such as Doppler ultrasound and CT pulmonary angiography, are essential for timely diagnosis and treatment of thrombosis in this population^[6,7]^. Despite the recognized thrombotic risk, management strategies for thrombosis in HIV and SCD co-infected individuals remain suboptimal, as there are no standardized guidelines for this complex patient group. Anticoagulation therapy, which is the mainstay of thrombosis management, must be carefully tailored to each patient, considering both the risk of clot formation and the potential for bleeding complications. Antiretroviral therapy may also need to be adjusted to minimize drug interactions with anticoagulants. The optimal treatment approach requires coordination between hematologists, infectious disease specialists, and cardiologists, ensuring that both the thrombotic risk and the underlying diseases are appropriately addressed. There is also a need for more research into specific thrombosis prevention strategies and the role of disease-modifying therapies for SCD in reducing thrombotic complications^[8-10]^.

## Aim

The aim of this review is to critically examine the pathophysiology, clinical manifestations, risk factors, and management strategies for thrombosis in individuals with HIV and SCD co-infection.

## Pathophysiology of thrombosis risk in SCD and HIV co-infection

**Figure 1.**
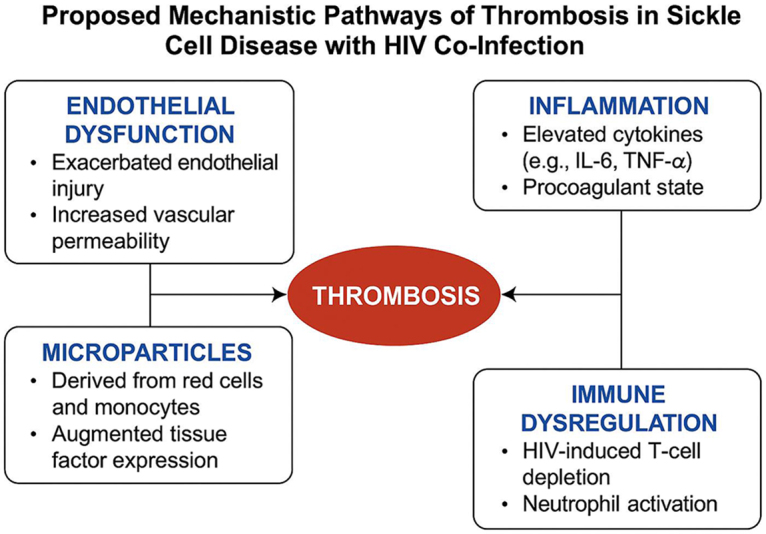
Proposed mechanistic pathways of thrombosis in sickle cell disease with HIV co-infection.

The pathophysiology of thrombosis risk in individuals with both SCD and HIV co-infection is complex and multifactorial, with both conditions independently contributing to a heightened thrombotic state. The interaction between these two diseases results in a synergistic effect, exacerbating the thrombotic risk and complicating clinical management^[10]^. In SCD, the primary driver of thrombosis is the abnormal sickling of red blood cells under low oxygen conditions. This process leads to mechanical damage to the blood vessels and endothelium, which triggers the release of procoagulant factors. The sickled cells also increase blood viscosity and promote the activation of platelets and the coagulation cascade. The resulting endothelial injury further contributes to vascular inflammation, making the blood vessel walls more prone to thrombosis. Additionally, vaso-occlusive events, which are hallmark features of SCD, increase the risk of DVT and PE. These events cause both local and systemic hypercoagulability, which predisposes patients to thrombotic complications. In individuals with SCD, both venous and arterial thrombosis are common, with stroke being a significant concern, particularly in pediatric patients^[11–14]^. In HIV, chronic immune activation and inflammation are key contributors to the increased thrombotic risk. HIV directly affects endothelial cells through viral binding to receptors such as CCR5 and CXCR4, leading to endothelial dysfunction. This dysfunction is characterized by a reduction in the production of nitric oxide, which normally helps to maintain vascular tone and prevent clot formation. Additionally, HIV infection promotes an inflammatory state by increasing the levels of pro-inflammatory cytokines such as TNF-α, IL-6, and IL-8, which further activate endothelial cells and platelets, contributing to the coagulation process. HIV infection also induces a prothrombotic environment by altering lipid metabolism and increasing levels of coagulation factors such as fibrinogen and von Willebrand factor. These alterations in coagulation profiles are compounded by the effects of ART, which, depending on the regimen, can have both beneficial and adverse effects on thrombotic risk^[15,16]^. The co-occurrence of SCD and HIV exacerbates these mechanisms of thrombosis risk. For example, while SCD causes endothelial injury through sickle cell-induced blood flow abnormalities, HIV contributes to endothelial dysfunction through chronic inflammation. These two processes together lead to a vicious cycle of endothelial injury and thrombus formation. Additionally, patients with SCD often require blood transfusions, which can further increase the risk of thrombosis through hyperviscosity and iron overload. The combination of SCD-induced sickling, HIV-mediated endothelial dysfunction, and ART-related metabolic changes leads to an amplified prothrombotic state in these individuals. This heightened risk is further compounded by the use of antiplatelet or anticoagulant therapy, which must be carefully managed to avoid excessive bleeding risks in this patient population (Fig. 1)^[17–19]^.

## Clinical manifestations of thrombosis in SCD and HIV co-infection

The clinical manifestations of thrombosis in individuals with both SCD and HIV co-infection are varied and can often mimic or overlap with other complications of either condition, making diagnosis challenging. The thrombosis-related events in this population are multifactorial, as both SCD and HIV contribute to vascular damage, endothelial dysfunction, and coagulation abnormalities. The presentation of thrombosis can thus be subtle or masked by the symptoms of SCD crises, requiring heightened clinical awareness and careful assessment^[7]^.

Venous thrombosis: In individuals with SCD and HIV co-infection, DVT and PE are common manifestations of venous thromboembolism. DVT may present with classic symptoms such as swelling, pain, and redness in the affected limb, particularly in the lower extremities. However, in the context of SCD, these symptoms may overlap with vaso-occlusive crises, which also cause limb pain and swelling, thus complicating the clinical picture. The risk of PE, characterized by chest pain, shortness of breath, tachypnea, and hypoxia, is also elevated in this population due to the thrombotic nature of venous clots that can travel to the lungs. In HIV-infected individuals, the chronic inflammatory state may increase the likelihood of clot formation in the veins, leading to a higher risk of DVT and subsequent PE^[3,20,21]^.

Arterial thrombosis: Arterial thrombosis, including stroke, is a particularly concerning complication in individuals with both SCD and HIV co-infection. Sickle cell disease predisposes individuals to ischemic strokes, especially in children, due to the obstruction of blood vessels by sickled red blood cells. However, the added effect of HIV-related endothelial dysfunction and chronic inflammation increases the risk of both arterial clot formation and ischemic events. Symptoms of arterial thrombosis, such as sudden weakness or numbness, loss of coordination, difficulty speaking, or vision changes, may suggest a stroke or transient ischemic attack. These symptoms should be immediately evaluated, as early intervention is critical to prevent irreversible neurological damage. Additionally, arterial thrombosis may also contribute to cardiovascular events such as myocardial infarction, which can present with chest pain, shortness of breath, and diaphoresis^[22–24]^.

Organ-specific thrombosis: Thrombosis in SCD and HIV co-infected individuals can also affect specific organs, further complicating disease management. In SCD, splenic infarctions due to sickled cells obstructing blood flow are common, but HIV-related immunosuppression may exacerbate these events. The combination of endothelial dysfunction, impaired immune responses, and sickling can result in compromised organ function, including splenic infarction, renal injury, and hepatic involvement. For example, renal thromboembolic events may present with hematuria, renal colic, or even acute renal failure, particularly in individuals with concurrent HIV infection. In the liver, thrombosis of the hepatic veins, known as Budd-Chiari syndrome, can result in abdominal pain, ascites, and hepatomegaly^[1,25]^.

Exacerbation of sickle cell crisis: Another manifestation of thrombosis in SCD and HIV co-infected individuals is the potential exacerbation of sickle cell crises. Vaso-occlusive episodes that lead to pain crises can be complicated by thrombosis, leading to a more severe clinical presentation. Thrombosis may aggravate ischemia in tissues already prone to hypoxia, such as the bones, lungs, and spleen. As a result, patients may experience more intense pain, swelling, and organ dysfunction. The addition of thrombotic events to these crises can also prolong hospitalization and increase the risk of complications such as infection, bleeding, and multi-organ failure^[25–28]^.

Complications of antiretroviral therapy: ART, essential for managing HIV, may also contribute to thrombotic events in this co-infected population. Certain ART regimens, particularly those containing protease inhibitors or non-nucleoside reverse transcriptase inhibitors, have been associated with dyslipidemia, insulin resistance, and other metabolic changes that promote a prothrombotic state. These changes can increase the risk of cardiovascular and cerebrovascular events, including stroke and myocardial infarction. ART-induced metabolic disturbances can therefore exacerbate the risk of thromboembolic events in patients with SCD, further complicating their clinical management^[29]^.

Clinical challenges in diagnosis and management: The overlapping symptoms of thrombosis with other complications of SCD and HIV infection present significant diagnostic challenges. For example, pain, swelling, and redness associated with DVT may be confused with a vaso-occlusive crisis in SCD, while pulmonary embolism can be misinterpreted as a respiratory infection or an acute chest syndrome, a common complication in SCD. Furthermore, the anticoagulation therapy required for thrombosis management must be carefully balanced with the risk of bleeding in individuals with SCD, as excessive bleeding can result from the underlying hemolytic anemia and the use of anticoagulants. As such, a thorough diagnostic workup, including imaging techniques like Doppler ultrasound, CT scans, and MRI, is essential for accurate diagnosis^[30–32]^.

## Risk factors for thrombosis in HIV and SCD co-infection

The risk of thrombosis in individuals with both SCD and HIV co-infection is multifactorial, involving a combination of genetic, disease-specific, and treatment-related factors. Both SCD and HIV independently increase the risk of thromboembolic events, and their co-occurrence exacerbates this risk due to synergistic effects^^[33]^^. Below are the primary risk factors that contribute to thrombosis in this dual-diagnosis population:

### Endothelial dysfunction

Endothelial dysfunction is a significant factor in the pathogenesis of thrombosis in HIV and SCD co-infection. In SCD, the sickling of red blood cells causes physical damage to the endothelial cells lining the blood vessels. This damage promotes the release of procoagulant molecules and enhances the expression of adhesion molecules that facilitate platelet aggregation and clot formation. In HIV, chronic viral replication and immune activation directly affect the endothelial cells, leading to their dysfunction. HIV induces endothelial cell activation through pro-inflammatory cytokines, resulting in an increased procoagulant state. The combination of sickle cell-induced and HIV-mediated endothelial injury increases the risk of thrombosis, particularly in smaller vessels^[34]^.

### Chronic inflammation

Both SCD and HIV are associated with chronic inflammation, which plays a pivotal role in thrombosis risk. In SCD, repeated vaso-occlusive crises and hemolysis trigger the release of pro-inflammatory cytokines and activation of the coagulation cascade. This systemic inflammatory response contributes to increased levels of fibrinogen, von Willebrand factor, and other pro-thrombotic molecules. Similarly, HIV infection causes persistent immune activation and inflammation, with elevated levels of pro-inflammatory cytokines such as IL-6, TNF-α, and IL-8. Chronic inflammation not only activates the endothelial cells but also promotes platelet aggregation, clot formation, and a hypercoagulable state. The cumulative inflammatory burden in co-infected individuals significantly increases the likelihood of thrombotic events^[12]^.

### Coagulation abnormalities

Coagulation abnormalities in SCD and HIV are key contributors to thrombotic risk. In SCD, the sickling of red blood cells causes mechanical damage to the vasculature, which results in the release of procoagulant factors such as tissue factor and von Willebrand factor. These factors promote platelet aggregation and enhance the coagulation cascade, leading to hypercoagulability. Additionally, SCD patients often experience hemolysis, which releases free hemoglobin into the bloodstream. Free hemoglobin has been shown to inhibit nitric oxide, a vasodilator that protects against thrombus formation, thereby promoting clot formation. In HIV, the viral infection itself induces alterations in coagulation by increasing levels of procoagulant proteins and decreasing anticoagulant proteins. HIV-related coagulopathy also disrupts the fibrinolytic system, further promoting thrombosis. The combined effects of these coagulation abnormalities in both conditions create a hypercoagulable environment, significantly increasing the risk of thrombosis^[35]^.

### Antiretroviral therapy (ART)

The use of ART in HIV-positive individuals can influence thrombotic risk in complex ways. Some ART regimens, particularly those containing protease inhibitors (PIs) and non-nucleoside reverse transcriptase inhibitors (NNRTIs), have been linked to metabolic changes such as dyslipidemia, insulin resistance, and abdominal obesity, all of which contribute to a prothrombotic state. Protease inhibitors, in particular, can increase the levels of low-density lipoprotein cholesterol and triglycerides, which are risk factors for cardiovascular disease and thrombosis. Additionally, ART-induced lipodystrophy and fat redistribution can lead to increased central adiposity, which further exacerbates metabolic risk factors for thrombosis. These ART-related metabolic changes, coupled with the effects of HIV on coagulation, create an environment that increases the risk of both venous and arterial thromboembolic events in individuals with SCD and HIV co-infection^[36]^.

### Hyperviscosity syndrome in SCD

In individuals with SCD, the abnormal red blood cell morphology and increased blood viscosity significantly contribute to thrombotic risk. Sickle cells are less deformable and can aggregate, obstructing small blood vessels and promoting clot formation. During episodes of dehydration, infection, or hemolysis, blood viscosity can increase further, exacerbating the risk of thrombosis. This phenomenon is particularly problematic in individuals with HIV, as infections and immune activation are common in this population. The combination of sickle cell-induced blood flow abnormalities and HIV-related endothelial dysfunction leads to a heightened risk of thromboembolic events, particularly in the microvasculature^[37,38]^.

### Viral load and immune activation

High HIV viral load and immune activation are known to increase the risk of thrombosis in HIV-infected individuals. Elevated HIV viral load leads to increased levels of pro-inflammatory cytokines and activation of the immune system, which in turn promotes endothelial dysfunction and a hypercoagulable state. This effect is particularly concerning in individuals with HIV and SCD, as the immune activation associated with HIV infection exacerbates the already elevated inflammatory state seen in SCD. In addition, low CD4 counts, which are commonly observed in individuals with advanced HIV disease, are associated with an increased risk of thrombotic events. The combined impact of immune dysregulation from both HIV and SCD further increases the thrombotic burden in co-infected individuals^[39]^.

### Use of blood transfusions

Patients with SCD frequently require blood transfusions to manage complications such as anemia, stroke, or acute chest syndrome. While blood transfusions can improve oxygen delivery and reduce the risk of vaso-occlusion, they also increase the risk of thrombosis. Transfusions lead to an increase in blood volume and viscosity, which can exacerbate the thrombotic risk, especially in individuals with HIV. Additionally, repeated transfusions can result in iron overload, which has been shown to further contribute to endothelial dysfunction and thrombosis. The complex interplay between transfusion-related changes in blood rheology, sickle cell-induced vaso-occlusion, and HIV-related coagulopathy heightens the risk of thrombotic events in this population^[18]^.

## Management of thrombosis in HIV and SCD co-infection

The management of thrombosis in individuals with HIV and SCD co-infection presents unique challenges due to the complex interplay between both conditions. The combination of endothelial dysfunction, chronic inflammation, hypercoagulability, and metabolic alterations requires a multifaceted approach to both prevention and treatment.

### Pharmacologic management

#### Anticoagulation therapy

The cornerstone of thrombosis management is the use of anticoagulants, particularly in cases of DVT, PE, or stroke. In HIV and SCD co-infection, anticoagulation therapy must be carefully considered to balance the risk of bleeding with the prevention of further thrombotic events. The use of low-molecular-weight heparin (LMWH) is often preferred for initial treatment of acute thrombotic events due to its predictable pharmacokinetics and ease of use. For long-term management, warfarin or direct oral anticoagulants may be considered, but regular monitoring is essential to ensure therapeutic levels. The use of LMWH is favored in the acute setting due to its shorter half-life and more consistent anticoagulant effect. However, clinicians must be cautious about drug interactions between anticoagulants and ART, as some HIV medications, particularly PIs and certain NNRTIs, can affect the metabolism of anticoagulants, requiring dosage adjustments^[40]^.

#### Thrombolytic therapy

In life-threatening thrombotic events, such as massive pulmonary embolism or acute ischemic stroke, thrombolytic therapy may be considered. This therapy involves the use of drugs like tissue plasminogen activator to rapidly dissolve the clot. However, thrombolytic therapy carries a high risk of bleeding and must be used judiciously, especially in patients with hemolysis or recent surgical procedures. In individuals with SCD, where hemolysis may complicate bleeding risk, careful evaluation of the clot burden and overall clinical status is critical before initiating thrombolytic therapy^[41]^.

### Management of hypercoagulable state

#### Management of sickle cell disease

Managing the underlying sickle cell disease is crucial in preventing the progression of thrombosis. Hydroxyurea, an established treatment for SCD, can help reduce the frequency of vaso-occlusive crises and lower the risk of thrombotic complications by increasing fetal hemoglobin levels and reducing the overall sickling of red blood cells. Regular blood transfusions may also be used to reduce the viscosity of the blood and to lower the risk of sickling in critical conditions. These measures indirectly decrease thrombotic risk by improving oxygenation and blood flow, which are impaired in SCD due to the sickling of red blood cells. In patients with chronic pain or frequent crises, pain management and hydration are essential, as dehydration and inadequate oxygenation exacerbate the risk of thrombosis. During acute events, aggressive hydration and oxygen therapy are crucial to reduce blood viscosity and improve microvascular perfusion, which in turn lowers the risk of clot formation^[42]^.

#### Managing HIV infection

Effective management of HIV is essential to reducing thrombosis risk, as well-controlled viral load can minimize HIV-induced inflammation and endothelial dysfunction. ART is critical for viral suppression, and the choice of ART must be individualized, taking into account the potential interactions with anticoagulants. Some ART regimens, particularly those containing protease inhibitors or NNRTIs, may interact with anticoagulants, requiring close monitoring and dose adjustments. The goal of ART is to maintain a low viral load and optimize immune function, which in turn reduces immune activation and the systemic inflammation that contribute to hypercoagulability^[43]^.

### Lifestyle and supportive care

#### Lifestyle modifications

Lifestyle interventions can play an important role in managing thrombotic risk in HIV and SCD co-infection. Smoking cessation is crucial, as smoking exacerbates both SCD-related vascular damage and HIV-associated endothelial dysfunction. Exercise is also beneficial for improving cardiovascular health and reducing the risk of thrombosis. Regular, moderate-intensity physical activity can help improve blood flow and reduce hypercoagulability. However, patients with SCD should be advised against overexertion and dehydration, which could trigger a sickle cell crisis or increase the risk of thrombotic events. A balanced diet rich in fruits, vegetables, and low in saturated fats and salt can help reduce cardiovascular risk factors such as hypertension and dyslipidemia, which are common in HIV-positive individuals. Additionally, maintaining adequate hydration and avoiding dehydration is vital in reducing blood viscosity, especially in individuals with SCD^[44]^.

#### Monitoring and follow-up care

Patients with HIV and SCD require regular follow-up care to monitor for thrombotic events and adjust treatment plans accordingly. Routine screening for thrombotic complications such as DVT, PE, and ischemic stroke is recommended, especially in high-risk individuals. Regular blood tests to monitor hemoglobin levels, platelet counts, and coagulation markers (such as D-dimer and fibrinogen) are necessary to detect early signs of thrombosis or coagulopathy. Furthermore, cardiovascular health monitoring through echocardiograms and other imaging studies can help identify early signs of complications like pulmonary hypertension, which can contribute to thrombosis risk in this population^[45]^.

### Multidisciplinary approach

Managing thrombosis in HIV and SCD co-infection requires a multidisciplinary approach. Collaboration between hematologists, cardiologists, infectious disease specialists, and primary care providers is essential for comprehensive care. Additionally, integrating social work and mental health support is important for addressing the psychosocial challenges that these patients may face, including the emotional burden of living with multiple chronic conditions. Patients should be educated about the signs and symptoms of thrombosis, and be provided with support in adhering to both anticoagulation therapy and ART^[46,47]^.

## Conclusion

The co-occurrence of thrombosis risk in individuals with HIV and SCD presents a unique and complex challenge for healthcare providers. The interplay between HIV-induced endothelial dysfunction, chronic inflammation, hypercoagulability, and the vascular complications of SCD heightens the risk of thrombotic events. Effective management requires a multidisciplinary approach that integrates pharmacologic interventions such as anticoagulation therapy, management of hypercoagulability, and optimization of SCD and HIV treatments. Regular monitoring, lifestyle modifications, and supportive care are vital to minimize thrombotic complications and improve patient outcomes. Additionally, the careful balance between anticoagulation therapy and the potential bleeding risks associated with hemolysis and platelet dysfunction in SCD necessitates individualized care plans.

## Data Availability

Not applicable as this is a narrative review.

## Research registration unique identifying number (UIN)

Not applicable as this is a narrative review.
